# Robust Directional Angle Estimation of Underwater Acoustic Sources Using a Marine Vehicle

**DOI:** 10.3390/s18093062

**Published:** 2018-09-12

**Authors:** Jinwoo Choi, Jeonghong Park, Yoongeon Lee, Jongdae Jung, Hyun-Taek Choi

**Affiliations:** Korea Research Institute of Ships and Ocean engineering, Daejeon 34103, Korea; jwchoi@kriso.re.kr (J.C.); jeonghong@kriso.re.kr (J.P.); yglee@kriso.re.kr (Y.L.); jdjung@kriso.re.kr (J.J.)

**Keywords:** angle of arrival, direction of arrival, time difference of arrival, acoustic source localization, marine vehicle

## Abstract

Acoustic source localization is used in many underwater applications. Acquiring an accurate directional angle for an acoustic source is crucial for source localization. To achieve this purpose, this paper presents a method for directional angle estimation of underwater acoustic sources using a marine vehicle. It is assumed that the vehicle is equipped with two hydrophones and that the acoustic source transmits a specific signal repeatedly. The proposed method provides a probabilistic model for time delay estimation. The probability is recursively updated by prediction and update steps. The prediction step performs a probability transition using the angular displacement of the marine vehicle. The predicted probability is updated using a generalized cross correlation function with a verification process using entropy measurement. The proposed method can provide a reliable and accurate estimation of the directional angles of underwater acoustic sources. Experimental results demonstrate good performance of the proposed probabilistic directional angle estimation method in both an inland water environment and a harbor environment.

## 1. Introduction

An acoustic signal is one of the most useful pieces of information in an underwater environment, where the use of other types of sensors is limited: electromagnetic wave signals attenuate rapidly due to a high rate of absorption, and the use of optical sensors is limited due to water turbidity, but acoustic signals can easily travel over kilometers with low attenuation. Various acoustic sources transmit their own acoustic signal that can be used to identify and track them. For this reason, acoustic signals are widely used in underwater applications such as vehicle localization [1,2,3], communication [4,5], intruder surveillance [6,7] and so on. These underwater applications depend on localizing underwater acoustic sources, which is typically accomplished using a hydrophone array [8]. Angles of arrival (AOAs) are acquired using the time differences of arrivals (TDOA) and the location is estimated by fusing the AOAs with the least square solution [9].

The estimation of the AOA of an acoustic source has been studied by various researchers investigating sound source localization. Several TDOA-based sound source localization methods have been developed using various types of hydrophone arrays. Conventional methods, such as the generalized cross correlation (GCC) [10] and beamforming [11], are widely used to estimate AOAs. These methods determine the time delay that maximizes the similarity between a pair of acoustic signals. Maximum likelihood-based approaches have also been developed using expectation maximization or importance sampling [12,13]. To improve the resolution of time delay estimation methods, subspace-based approaches have been used. Smith et al. used a subspace-based estimation for underwater target tracking considering the Doppler effect [14]. When using hydrophone arrays in a close spacing, the phase difference has been used to estimate the direction of an acoustic source [15]. These acoustic source localization methods have also been used for speech recognition or verbal command of mobile robot systems in the air [16,17]. These methods all provide an estimate of the directional angle using only current acoustic data. Unfortunately, these methods can possibly provide inaccurate angular information when the current acoustic signal is affected by the multi-path effect or acoustic noise.

This paper proposes a method for the robust estimation of the AOAs of underwater acoustic sources. The proposed method estimates the directional angles of static underwater acoustic sources using a moving marine vehicle equipped with two hydrophones. Actual underwater equipment transmits signals repeatedly, periodically or non-periodically, so that the source can be identified using the characteristics of the acoustic signal. Therefore, the proposed method assumes that an acoustic source emits a specific signal repeatedly. The investigation of the characteristics of various acoustic sources based on their frequency bands is outside of the scope of this paper, which is focused on the robust estimation of the directional angle of general underwater acoustic sources with known frequency band information.

The proposed method estimates the directional angle of an underwater acoustic source using a Bayesian process [18]. A probabilistic model for time delay is formulated [19] and is updated whenever a new acoustic signal is detected. The update process consists of three steps. First, a prior probability is calculated using a state transition model and the previous probability. Angular displacement data acquired from an onboard inertial measurement unit (IMU) sensor on the marine vehicle are used for the state transition model. Second, GCC is used to acquire directional information from the current acoustic signal. The correlation function is used as the observation to inform the estimation of time delay probability. Finally, the utility of the current observation is verified by comparing the entropy of the current correlation with the entropy of the prior probability. The time delay probability is updated only once the current correlation exhibits a lower entropy than the prior probability.

The proposed method provides a well-organized framework for the estimation of the directional information of underwater acoustic signal sources, and can provide robust angular information even when presented with multipath and underwater acoustic noises.

The remainder of this paper is organized as follows. Section 2 describes the TDOA estimation problem and the formulation of the probabilistic time delay estimation. Section 3 presents the proposed method for directional angle estimation of the underwater acoustic source. Section 4 presents experimental results conducted in an inland water environment and in a harbor environment. Section 5 presents conclusions and future directions for the development of the proposed method.

## 2. Probabilistic Modeling of TDOA Estimation

### 2.1. TDOA Based Directional Angle Estimation

**Figure 1:**
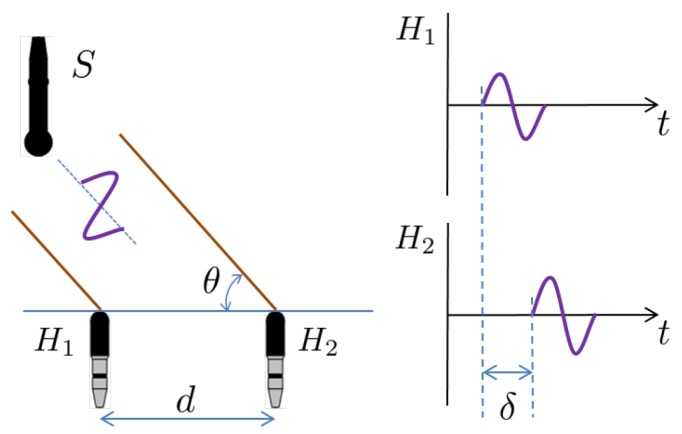
Directional angle estimation of underwater acoustic source using TDOA. Assuming that the source (*S*) is located a large distance from the receivers ($H_{1}$ and $H_{2}$), the plane wave arrives at the receivers at measurably different times.

The proposed method uses a marine vehicle equipped with two hydrophones to estimate the directional angle of an underwater acoustic source. Figure 1 shows the principle of the proposed TDOA-based direction estimation method. When a source transmits an acoustic signal, two hydrophones receive the same signal separated by a time delay (δ). Assuming planar far-field propagation of the acoustic wave, the direction of the acoustic source (θ) can be acquired as

(1) θ=acos(δ·c/d),

where *c* and *d* are the sound velocity underwater and the spacing between the two hydrophones, respectively. All the variables except the time delay δ in Equation (1) can be regarded as known values. Therefore, acquiring an accurate time delay between two acoustic signals is essential to estimate the directional angle of an acoustic source.

### 2.2. Defining the Probabilistic Model of Time Delay

The proposed method estimates the time delay using a probabilistic model to acquire the directional angle of an acoustic source. Assuming that an acoustic source transmits the same signal repeatedly, the directional angle can be estimated probabilistically by using a history of the estimation. For this purpose, the probabilistic model, $P(\delta_{t}=\delta_{k})$, is formulated to estimate the time delay. The function $P(\delta_{t}=\delta_{k})$ represents the probability that the time delay at time *t* is $\delta_{k}$. This is calculated by a Bayesian update process using both state transition models and observations, as in Equation (2) [18] (pp. 24–32).

(2) $$\begin{array}{cc} \;P(\delta_{t}=\delta_{k}|z_{1:t},u_{1:t}) & =\eta_{1}\cdot P\left(z_{t}|\delta_{t}=\delta_{k},z_{1:t-1},u_{1:t}\right)P\left(\delta_{t}=\delta_{k}|z_{1:t-1},u_{1:t}\right)\\ & =\eta_{1}\cdot P\left(z_{t}|\delta_{t}=\delta_{k}\right)P\left(\delta_{t}=\delta_{k}|z_{1:t-1},u_{1:t}\right), \end{array}$$

where $\eta_{1}$ is a normalizing factor, and $u_{1:t}$ and $z_{1:t}$ are the state transition models and observations, respectively, from time 1 to *t*. The middle part of Equation (2) is the likelihood acquired by current observation and the final part is the prior probability, acquired from the state transition model and the previous probability. Using the probability, the time delay is determined as the value that possesses the maximum probability,

(3) $$\begin{array}{c} \delta =\underset{\delta_{k}}{\arg\;\max}P\left(\delta_{t}=\delta_{k}\right). \end{array}$$

The probability of time delay is re-calculated when a new acoustic signal is detected, then the directional angle of the acoustic source is estimated by the corresponding observed time delay.

## 3. Time Delay Estimation Using Bayesian Probability

The time delay probability is recursively calculated by prediction and update processes. The prediction step performs a probability transition to acquire the prior probability using the state transition model and previous probability. The update step is achieved by re-calculating the time delay probability using the current observation. The following subsections describe these processes in detail.

### 3.1. Angular Displacement as the State Transition Model

The prediction step requires a state transition model for the probability transition representing the acquisition of the predicted time delay probability in the current time step from the probability of the previous time steps. For this purpose, the angular displacement measured by the onboard IMU sensor of the marine vehicle is used in the proposed method. The AOA of an acoustic source changes with both the translational and angular motion of the vehicle. However, the proposed method was developed for a passive localization system, so the exact location of the acoustic source with respect to the marine vehicle is not available due to the absence of range information. Alternatively, the angular displacement of the marine vehicle can be used as the state transition model. When an acoustic source is located far from the vehicle (as assumed in this study), the angular motion of the vehicle has a more dominant effect on the change in the AOA than the translation of the vehicle. Therefore, the angular displacement is used in the current application as the state transition model and the effect of the translation is reflected in the Gaussian noise model.

The prior probability using the angular displacement can be derived by the total probability theorem [18] (pp. 16–77) as in Equation (4):

(4) $$\begin{array}{cc} P(\delta_{t}=\delta_{k}|z_{1:t-1},u_{1:t}) & =\sum_{i}P\left(\delta_{t}=\delta_{k}|\delta_{t-1}=\delta_{i},u_{1:t},z_{1:t-1}\right)P\left(\delta_{t-1}=\delta_{i}|u_{1:t-1},z_{1:t-1}\right). \end{array}$$

The first part of Equation (4) can be obtained from the state transition model of angular displacement (dψ) as:

(5) $$\begin{array}{cc} P(\delta_{t}=\delta_{k}|\delta_{t-1},d\psi_{1:t},z_{1:t-1}) & =P(\delta_{t}=\delta_{k}|\delta_{t-1},d\psi_{1:t}), \end{array}$$

(6) $$\begin{array}{c} =N\left(d\psi ;{\hat{d\psi }}_{imu},\sigma_{d\psi }^{2}\right){|}_{d\psi =\theta_{ik}}, \end{array}$$

where N is the normal distribution function, ${\hat{d\psi }}_{imu}$ is the angular displacement measured by the IMU, and

(7) $$\begin{array}{c} \theta_{ik}=acos\left(\delta_{k}\cdot C/d\right)-acos\left(\delta_{i}\cdot C/d\right). \end{array}$$

The last part of Equation (4) is obtained from the posterior probability at the previous time step t−1.

The probability for every effective time delay can be calculated using this state transition model, and the prediction step provides the prior probability of Equation (2). This process allows the proposed method to estimate the directional angle using not only the current acoustic signal, but also the history of its estimation.

### 3.2. Generalized Cross Correlation as the Observation Model

The measurement model of the Bayesian process is the observation resulting from the current sensor data. The proposed method adopts GCC to acquire the measurement of time delay probability. Currently, GCC is one of the most popular methods for the estimation of the time delay between two signals. It is easy to implement and has low computational burden, making real-time estimation possible.

The process of GCC is executed only when a target signal is detected. Here, the target signal is an acoustic signal transmitted by the target source. The target signal is extracted from the raw acoustic signal using a band-pass filter. The GCC is then performed on the extracted signal by the following procedure.

1. Acquire target signals, $x_{1}\left(t\right)$ and $x_{2}\left(t\right)$, from two hydrophones.
2. Apply a Fourier transform to the acquired signals, as:
  (8) $$\begin{array}{c} X_{i}\left(w\right)=\int_{-\infty }^{\infty }x_{i}\left(t\right)e^{-jwt}dt. \end{array}$$
3. Acquire the cross spectrum by multiplying $X_{1}\left(w\right)$ and the complex conjugate of $X_{2}\left(w\right)$:
  (9) $$\begin{array}{c} S_{x_{1}x_{2}}\left(w\right)=X_{1}\cdot X_{2}^{*}. \end{array}$$
4. Acquire the cross correlation by an inverse Fourier transform, multiplying it by a weighting function Ψ(w):
  (10) $$\begin{array}{c} R_{x_{1}x_{2}}\left(\tau \right)=\int_{-\infty }^{\infty }\Psi \left(w\right)S_{x_{1}x_{2}}\left(w\right)e^{jw\tau }dw. \end{array}$$
  Here, the weighting function Ψ(w) is used in accordance with the PHAT (Phase Transform) function in the proposed method in Equation (11).
  (11) $$\begin{array}{c} \Psi_{PHAT}\left(w\right)=\frac{1}{|X_{1}\left(w\right)X_{2}^{*}\left(w\right)|}. \end{array}$$

Using the cross correlation function $R_{x_{1}x_{2}}\left(\tau \right)$ as an observation in Equation (2), the proposed method can calculate the current time delay probability, enabling it to estimate the directional angle of the acoustic source from the time delay value that possesses the maximum probability.

### 3.3. Filtering Out False Direction Estimates

The correlation function from GCC is used as a measurement model to provide the update of the time delay probability. Unfortunately, cross correlation always has the potential for inaccurate time delay estimates due to various underwater acoustic noise sources. Even though the Bayesian process can alleviate the effect of a noisy observation using the state transition model, filtering out noisy data in advance before the update step helps to improve the performance of the directional angle estimation. For this purpose, the proposed method performs an additional evaluation process of the current observation using the entropy of probability.

The entropy, *H*, of probability *P* is defined as

(12) $$\begin{array}{c} H\left(P\right)=\sum_{i=1}^{n}-P\left(i\right)log_{n}P\left(i\right), \end{array}$$

where *n* is the total number of effective time delay values in the probability set. Here, an effective time delay value is defined as that which results in a real value for the directional angle, as determined by Equation (1), and thus *n* is determined by Equation (13).

(13) $$\begin{array}{c} n=2\times \left\lfloor d/c\cdot f_{s}\right\rfloor +1, \end{array}$$

where $f_{s}$ represents the sampling frequency of the acoustic signal acquisition system.

The entropy of a probability reflects the amount of information contained in the probability. In other words, a more informative probability possesses less entropy. The proposed method compares the entropies of measurement (the cross correlation of the current acoustic signal) with those of the prior probability. When the current measurement is affected by noisy data, it will possess a large entropy value. Otherwise, it will possess a small entropy value, making the posterior probability more informative through the update process. Using this characteristic of entropy, only the observations with smaller entropy than the prior probability are used to update the time delay probability:

(14) $$\begin{array}{c} H\left(P_{cc}\right)>H\left(P_{prior}\right), \end{array}$$

where $P_{cc}$ is the measurement acquired from cross correlation function (Equation (10)), and $P_{prior}$ is the prior probability acquired in the prediction step using the state transition model (Equation (4)).

**Figure 2:**
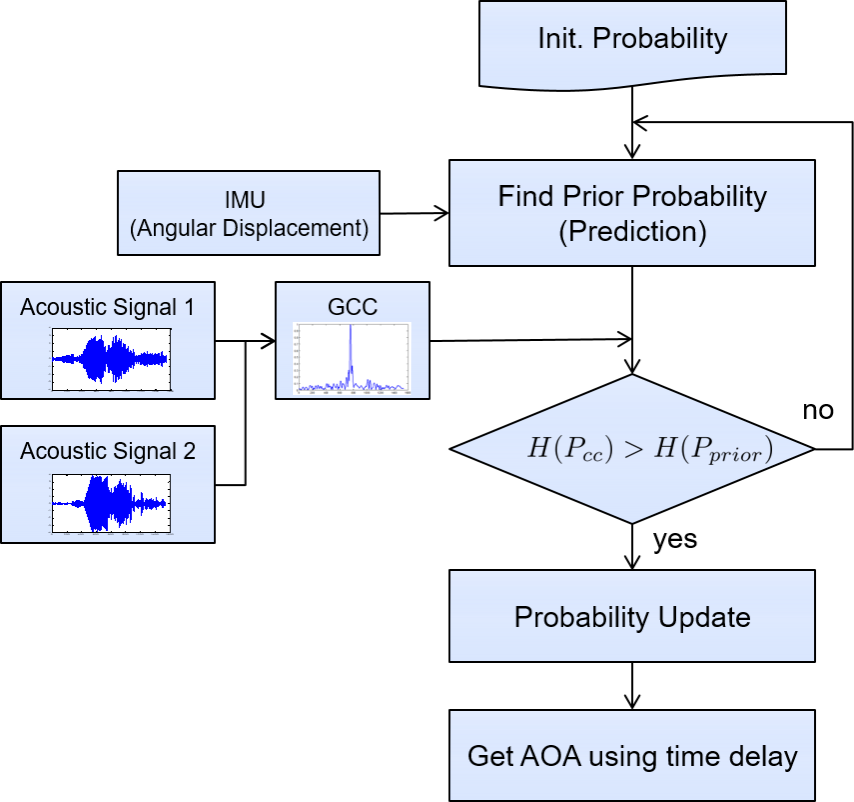
A flowchart for the estimation of directional angle using a marine vehicle equipped with two hydrophones.

Figure 2 depicts a flowchart for the overall process of the proposed method. As shown, observations that do not satisfy Equation (14) are regarded as noisy and the prediction step is performed without this noisy data.

**Figure 3:**
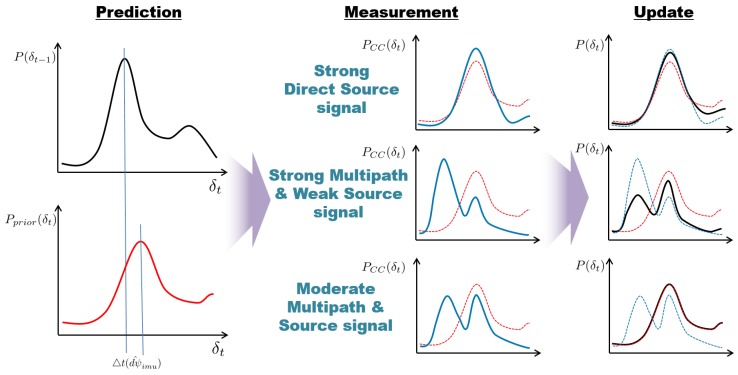
Illustration of time delay probability calculation. The black and red lines in the prediction step represent the probability at time step t−1 and the predicted prior probability calculated by the angular displacement of the marine vehicle at time step *t*, respectively. In the measurement and update steps, the red lines represent prior probability, the blue lines represent the cross-correlation function, and the black lines represent the calculated time delay probability at time step *t*.

Figure 3 illustrates how the proposed method addresses three different cases of acoustic signal measurement: a strong direct source signal, strong multipath and weak source signal, and moderate multipath and source signal. The proposed method first calculates the prior probability (red line) from previous probability (black line) using the angular displacement of the marine vehicle (${\hat{d\psi }}_{imu}$) in the prediction step. The time delay probability is shifted by the corresponding time delay ($▵t({\hat{d\psi }}_{imu})$) and widened due to the Gaussian noise. Both the proposed method and GCC can adequately address the case of a strong direct source signal. When the multipath effect is much stronger than the direct source signal, the proposed method inevitably updates the probability using the weak measurement because it is difficult to determine whether the measurement is caused by multipath. In this manner, the effects of the occasional multipath signal can be alleviated by the prior probability of the prediction step, as shown in the updated result for this case. If the multipath effect is not repeatedly significantly stronger than the direct signal, the proposed method will recover the correct time delay through adjustments to the probability from subsequent measurements. In the case of moderate multipath and source signal in which the strengths of the multipath and direct signals are similar, the measurement is likely to be neglected because of the entropy test step. Consequently, the location would be calculated using only the prior probability. Taken together, the processes constituting the proposed method enable it to adequately handle the presence of a non-repetitive multipath effect.

The proposed method provides a well-structured framework for the estimation of the directional angle of a repetitively transmitting acoustic source. Through the above processes, the proposed method can provide a robust result even when current observations are affected by noisy data or reflective acoustic waves.

## 4. Experimental Results of Directional Angle Estimation

Two experiments were conducted to verify the proposed method using an unmanned surface vehicle (USV) equipped with two B & K 8103 hydrophones (Brüel & Kjær, Nærum, Denmark) that provided omnidirectional response and a B & K Nexus pre-amplifier (Brüel & Kjær, Nærum, Denmark). The receiving sensitivity of the hydrophones was −211 dB re 1 V/μ Pa. Details of the hydrophones and pre-amplifier can be found in [20]. The spacing between the two hydrophones was 0.6 m. The USV was equipped with onboard DGPS (Differential Global Positioning Systems) (SMART6 from Novatel (Calgary, AB, Canada) [21]) and IMU (3DM-GX3-25 from MicroStrain (Williston, VT, USA) [22]) sensors. Note that the proposed method can be applied to any type of marine vehicle, including autonomous underwater vehicles (AUVs) or remotely operated vehicles (ROVs). In this study, the USV was used for its simplicity in the experimental implementation and the ease of obtaining GPS data to assess the accuracy of the location estimates.

### 4.1. Inland Water Environment Experiment

**Figure 4:**
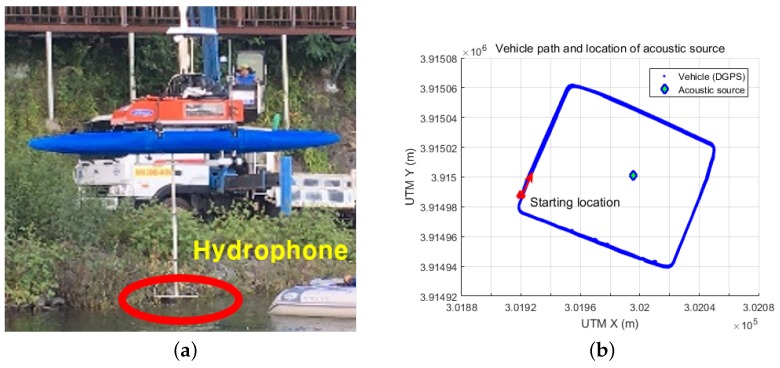
Experimental setup: (**a**) USV equipped with two hydrophones; and (**b**) vehicle path and location of acoustic source acquired from DGPS.

The first experiment was conducted in an inland water environment. A single acoustic source was moored in place using a buoy system consisting of a B & K 8105 hydrophone [20] connected to a signal generator that repeatedly transmitted a pre-defined, 20 ms long chirp signal of 30–55 kHz, five times per second. The USV followed a set rectangular path (of about 110 m × 90 m) that rotated it around the source. Figure 4b shows the path of the USV and the location of the acoustic source, as acquired from DGPS. The red triangle represents the starting location of the USV, which finished the experiment near its starting location. The vehicle moved along the path three times over almost 2000 s. The distance between the vehicle and the source varied from 20 m to 80 m. The signal-to-noise ratio (SNR) in the experimental environment was measured in the range of 8–23 dB.

**Figure 5:**
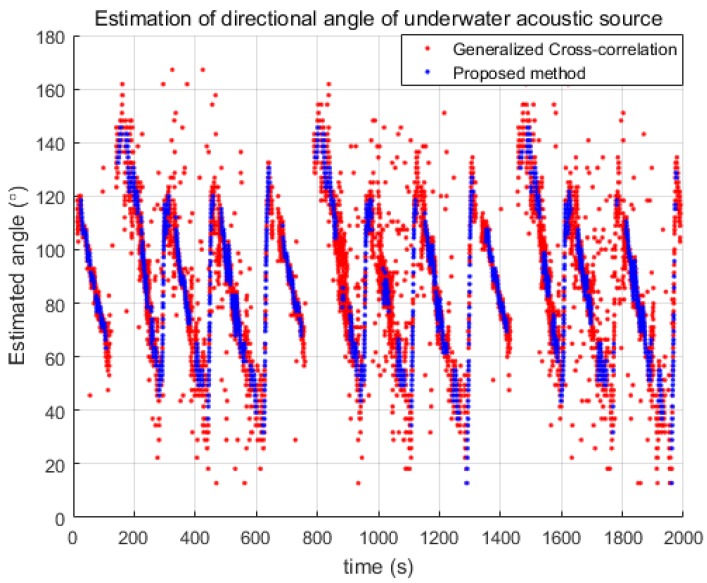
Estimated directional angles using generalized cross correlation and the proposed probabilistic method.

The USV estimated the directional angle of the acoustic signal acquired at a 100 kHz sampling rate with the results shown in Figure 5. The red dots represent the result of the GCC and the blue dots represent the estimated angles of the proposed probabilistic estimation. It can be seen in the figure that the GCC resulted in many outlier directional angles even though the overall trends are similar for both methods. The proposed probabilistic method clearly provided a reliable and robust estimation of the directional angle. Most of the outliers can be observed to have been filtered out by the proposed state transition model and entropy method.

**Figure 6:**
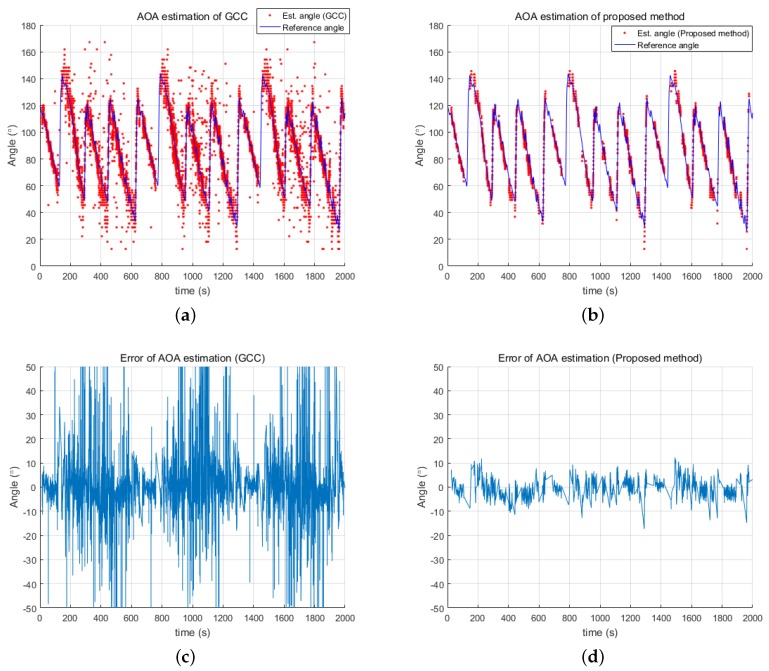
Error of estimated directional angles: (**a**) reference angle and estimated angle from GCC; (**b**) reference angle and estimated angle from the proposed method; (**c**) error of GCC-estimated angle; and (**d**) error of estimate from the proposed method.

Figure 6 provides the accuracy of the proposed method by comparing the reference angles, considered to be the correct values, acquired from the DGPS location of the acoustic source and from the location and the heading of the USV, acquired from DGPS and magnetic compass readings. In Figure 6a,b, the blue line represents the reference angle and the red dots represent the angle estimated by the GCC and the proposed method, respectively. As shown in Figure 6b, the angles estimated by the proposed method fit the reference angles well. The error between the reference angles and the estimated angles is shown in Figure 6d to have been maintained within $\pm 10^{\circ }$ for most cases. On the other hand, the GCC produced much larger errors, as shown in Figure 6c, due to the outliers. These results demonstrate the robustness and accuracy of the proposed method over the conventional GCC method.

### 4.2. Harbor Environment Experiment

**Figure 7:**
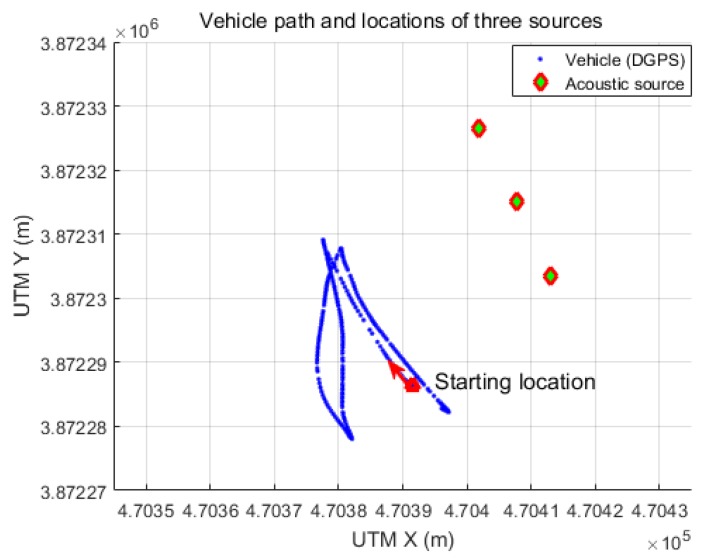
Vehicle path and locations of three acoustic sources acquired from DGPS.

The second experiment was conducted in a harbor environment. In this experiment, the same USV was used as in the first experiment, but three acoustic sources were simultaneously tracked. The three acoustic sources transmitted chirp signals in different frequency bands of 25–30 kHz, 30–35 kHz, and 35–40 kHz. The signal length and transmission period were the same as in the inland experiment. Three different band-pass filters were used to extract the target signals transmitted by the three sources. Figure 7 shows the experimental setup. The three acoustic sources were mounted on the harbor structure and the USV was moved along the path shown in the figure. The vehicle traveled a total distance of about 120 m, and the distance between the vehicle and the sources ranged from 30 to 50 m. The SNR in the experimental environment was measured in the range of 3–21 dB.

**Figure 8:**
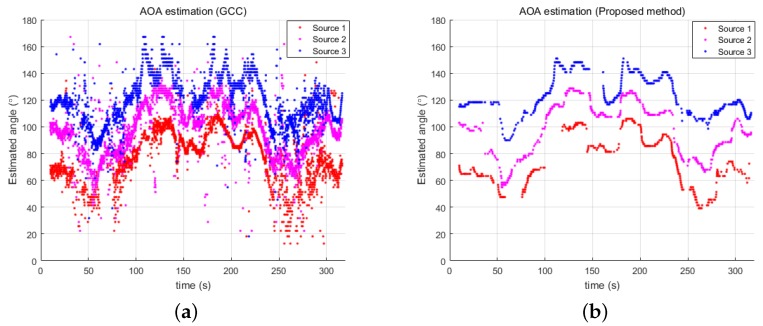
Estimated directional angles of three acoustic sources by: (**a**) GCC; and (**b**) the proposed method.

Figure 8 provides the estimated directional angles of the three underwater acoustic sources using the GCC and proposed methods. Figure 8a shows that the experimental result from GCC involves many outliers, so the estimated directional angles are very noisy. The proposed probabilistic estimation of the directional angles filtered out these noisy data and provided a reliable result, as shown in Figure 8b.

**Figure 9:**
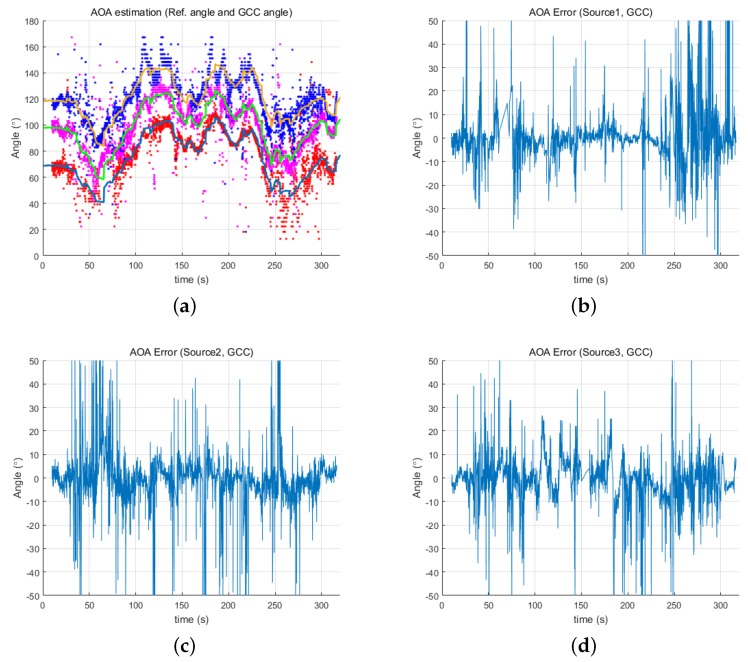
Error of angles determined by GCC: (**a**) reference angles and estimated angles for three acoustic sources from GCC; (**b**) error of Source 1; (**c**) error of Source 2; and (**d**) error of Source 3.

**Figure 10:**
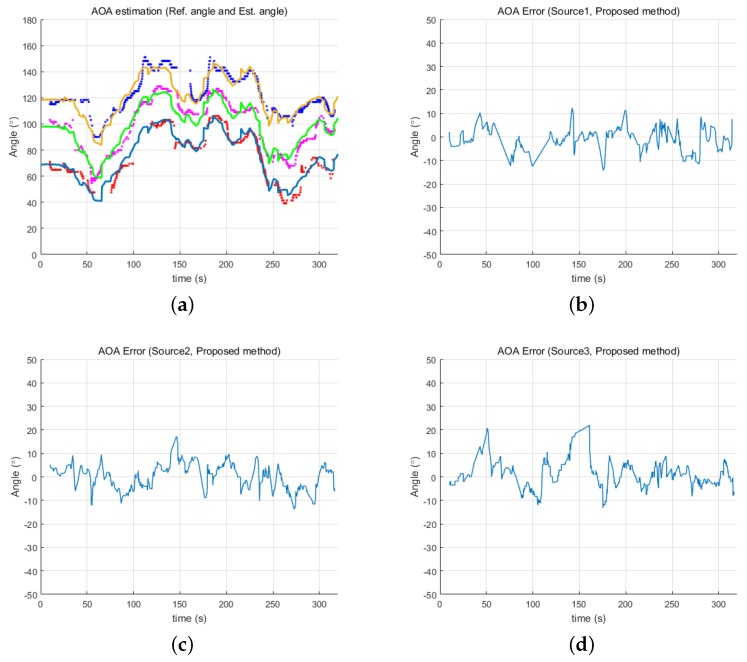
Error of angles determined by the proposed method: (**a**) reference angles and estimated angles of three acoustic sources from the proposed method; (**b**) error of Source 1; (**c**) error of Source 2; and (**d**) error of Source 3.

Figure 9 and Figure 10 show the estimated angles from both the GCC and proposed methods, respectively, in relation to the reference angles. The proposed method resulted in a more accurate estimation than GCC, providing much less error for all three underwater acoustic sources. Although the estimation error of the proposed method was still less than that of GCC, the error level was slightly larger than in the first experiment as there are generally more acoustic noise sources in an ocean environment than in an inland water environment. These noise sources can affect the resulting estimates. Furthermore, the simultaneous transmission of three acoustic signals might also cause errors in the estimates. Despite these detrimental effects, the proposed method provided a directional angle estimate within $\pm 15^{\circ }$ for most of the experiment.

These experimental results confirm the performance of the proposed AOA estimation method in both an inland and an ocean environment. The proposed method has been demonstrated to provide reliable and accurate estimates even with noisy acoustic data by using the state transition model and entropy measurement in the Bayesian update process.

## 5. Conclusions

This paper describes a method for the estimation of the directional angle of underwater acoustic sources. The proposed method was developed for a marine vehicle equipped with two hydrophones and was found to provide a reliable estimation of directional angles even with the noisy acoustic signals in an underwater ocean environment. The proposed method performs a probabilistic estimation of time delay that was formulated and calculated using a Bayesian update process. The prediction step is accomplished using the angular displacement of the vehicle as a state transition model. In the update step, the cross correlation function of GCC is adopted to provide the measurement. Then, an entropy comparison is used to verify the utility of the current measurement. Thorough these processes, the proposed method is able to estimate the directional angles of underwater acoustic sources reliably and accurately. The performance of the proposed method was confirmed by two experiments, one conducted in inland water environment and one in a harbor environment.

The proposed method provides a well-organized structure for the directional angle estimation of underwater acoustic sources, and can be applied in any type of marine vehicle (e.g., AUV or ROV). Although the proposed method was only implemented with GCC as a measurement model in this study, any other method that provides time delay estimation could be used as the measurement model. By using the proposed method as a basic framework, the estimation of directional angle by any time delay estimation method could be achieved. Furthermore, the proposed directional angle estimation method can be applied to a variety of underwater applications, such as noise source tracking or autonomous navigation of underwater vehicles. The problem of tracking a moving acoustic source might also be an interesting research topic as an extension of the proposed method.

## Abbreviations

The following abbreviations are used in this manuscript:

TDOA	Time Difference of Arrival
AOA	Angle of Arrival
GCC	Generalized Cross Correlation
IMU	Inertial Measurement Unit
DGPS	Differential Global Positioning Systems
SNR	Signal-to-Noise Ratio
